# Characterization of the canine *CLCN3 *gene and evaluation as candidate for late-onset NCL

**DOI:** 10.1186/1471-2156-7-13

**Published:** 2006-03-03

**Authors:** Anne Wohlke, Ottmar Distl, Cord Drogemuller

**Affiliations:** 1 Institute for Animal Breeding and Genetics, University of Veterinary Medicine Hannover, Bünteweg 17p, 30559 Hannover, Germany; 2 Institute of Genetics, Vetsuisse, University of Berne, Bremgartenstrasse 109a, 3001 Berne, Switzerland

## Abstract

**Background:**

The neuronal ceroid lipofuscinoses (NCL) are a heterogenous group of inherited progressive neurodegenerative diseases in different mammalian species. Tibetan Terrier and Polish Owczarek Nizinny (PON) dogs show rare late-onset NCL variants with autosomal recessive inheritance, which can not be explained by mutations of known human NCL genes. These dog breeds represent animal models for human late-onset NCL. In mice the chloride channel 3 gene (*Clcn3*) encoding an intracellular chloride channel was described to cause a phenotype similar to NCL.

**Results:**

Two full-length cDNA splice variants of the canine *CLCN3 *gene are reported. The current canine whole genome sequence assembly was used for gene structure analyses and revealed 13 coding *CLCN3 *exons in 52 kb of genomic sequence. Sequence analysis of the coding exons and flanking intron regions of *CLCN3 *using six NCL-affected Tibetan terrier dogs and an NCL-affected Polish Owczarek Nizinny (PON) dog, as well as eight healthy Tibetan terrier dogs revealed 13 SNPs. No consistent *CLCN3 *haplotype was associated with NCL.

**Conclusion:**

For the examined animals we excluded the complete coding region and adjacent intronic regions of canine *CLCN3 *to harbor disease-causing mutations. Therefore it seems to be unlikely that a mutation in this gene is responsible for the late-onset NCL phenotype in these two dog breeds.

## Background

Neuronal ceroid lipofuscinoses (NCL) represents a group of heritable neurodegenerative storage diseases in man, mice, and several domestic animals like cattle, sheep, goat, cat, and certain dog breeds [1]. NCL diseases are characterized by the accumulation of autofluorescent cytoplasmic storage bodies in cells of the brain and retina. NCL diseases cause neurological symptoms that progress relentlessly and culminate in a vegetative state in humans and premature death [2]. Canine late-onset NCL variants primarily affect Tibetan Terrier and Polish Owczarek Nizinny (PON) dogs. A monogenic autosomal recessive mode of transmission was suggested for those breeds [3,4]. NCL-affected dogs represent valuable animal models to study human late-onset NCL variants since human families segregating for adult NCL are infrequent. Human NCL is a genetically heterogeneous disease with six identified disease genes (*PPT1, TPP1, CLN3, CLN5, CLN6 *and *CLN8*) [5]. Causal mutations within the canine orthologs of the six known human NCL genes have not been identified in NCL-affected Tibetan Terrier and PON dogs [6-9]. Single point mutations in the coding regions of the canine *CLN8 *and *CLN5 *genes were found in affected English Setter and Border collie dogs, respectively, showing juvenile NCL [6,10]. There are still undiscovered loci causing NCL beside the six known human genes, as indicated by findings in NCL-affected domestic and laboratory animals. In White Swedish Landrace sheep a *CTSD *mutation was reported and a mutation within the ortholog canine *CTSD *gene was detected in NCL-affected American Bulldogs [11]. Recently, *CTSD *was excluded as candidate gene in NCL-affected Tibetan Terrier and PON dogs [12]. In mice the chloride channel 3 gene (*Clcn3*) encoding an intracellular chloride channel was described to cause a phenotype similar to NCL [13]. *Clcn3*-deficient mice are characterized by developmental retardation and higher mortality combined with neurological manifestations such as blindness, motor coordination deficit, and spontaneous hyperlocomotion similar to human and canine NCL. To evaluate whether the *CLCN3 *gene is involved in the NCL-affected Tibetan Terrier and PON dogs, we determined the full-length cDNA sequence, characterized the gene structure, and analyzed the coding sequence of the canine ortholog.

## Results and discussion

### Sequence analysis

**Figure 1:**
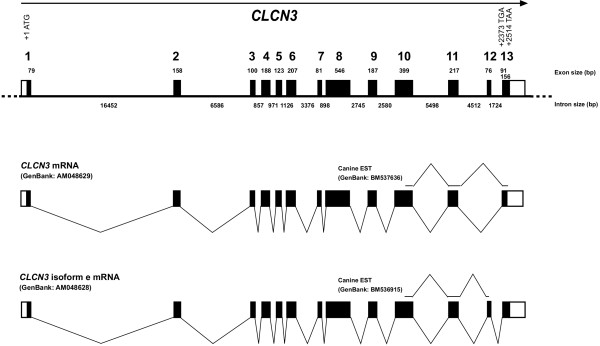
**Genomic structure of the canine *CLCN3 *gene**. Translated exons are shown as solid boxes, untranslated regions of exons as shaded boxes. The two alternative gene transcripts are shown below.

RT-PCR from canine lung mRNA amplified two splice variants, which were verified by direct DNA-sequencing of the RT-PCR products. Similar to the human *CLCN3 *sequence the alternative usage of exon 12 produces the shorter *CLCN3 *and the longer *CLCN3 *isoform e, respectively (Figure 1). Overlapping canine cDNA fragments containing all junctions between the exons were generated by RT-PCR, sequenced, and used for comparison with the genomic sequence. These analyses indicated that the canine *CLCN3 *gene consists of 13 exons separated by twelve introns. The canine *CLCN3 *gene spans 52 kb (Figure 1) compared to 14 exons over 100 kb in human *CLCN3 *(NCBI build 35.1)) because in dog no untranslated 5'-exon is used. All splice donor/splice acceptor sites conform to the GT/AG rule. The experimentally verified existence of the two alternative splice variants is in agreement with the initially identified canine 5'-EST sequences (Figure 1). In dog the shorter *CLCN3 *transcript [EMBL:AM048629] contains an open reading frame of 2,376 bp encoding a protein of 791 amino acids. The longer canine *CLCN3 *isoform e transcript [EMBL:AM048628] contains an open reading frame of 2,517 bp encoding a sequence of 838 amino acids.

### Mutation analysis

A total of 13 sequence polymorphisms (Table 1) were found in the examined dogs as compared to the *CLCN3 *reference sequence of the current dog genome assembly (boxer genome assembly 1.1). The codon in exon 2 with the A/G transition codes for glycine in both SNP variants and the codon in exon 9 with the A/G transition codes for proline in both SNP variants. None of the 11 intronic polymorphisms did affect splice sites in the *CLCN3 *gene. For the single PON dog there were seven polymorphisms compared to the Boxer reference sequence (Table 1).

**Table 1:** Polymorphisms and observed haplotypes in the canine *CLCN3 *gene

Position^1^	Boxer^2^	Haplotype	Tibetan Terrier						PON	
			1	2	3	4	5	6	1	2
intron 1	16409	T	T	T	T	T	T	T	T	C
exon 2	2	A	A	A	A	A	A	G	A	A
intron 2	3	G	A	G	G	A	G	A	G	G
intron 5	10	G	A	A	A	A	A	A	A	A
intron 6	40	A	A	A	A	A	A	A	del	del
intron 6	2861	T	T	T	C	C	T	T	T	T
intron 6	3061	C	G	G	G	G	G	C	G	G
intron 6	3266	G	A	A	A	A	A	G	A	A
intron 6	3270	G	A	G	G	A	A	A	G	G
intron 6	3291	C	T	T	T	T	T	C	T	T
intron 7	38	G	G	A	G	G	G	G	G	G
intron 7	144	C	T	C	T	T	T	T	C	C
exon 9	6	A	G	G	G	G	G	G	G	G

^1 ^Numbering refers to the position of the polymorphic nucleotide within the given exon or intron respectively.

^2 ^Nucleotide refers to the publicly available dog genome sequence (AAEX01020012) from the boxer named Tasha.

Assuming linkage disequilibrium between the polymorphic loci six different haplotypes could be constructed for the SNP genotypes in the Tibetan Terrier dogs (Table 1). Four out of six haplotypes occurred in both, affected and unaffected dogs, respectively (Table 2). Only the haplotypes 3 and 4 occurred in a single heterozygous NCL-affected Tibetan Terrier dog (Table 2). Due to the assumption of a single recessive founder mutation within this breed we expect homozygosity in affected individuals. Table 2 summarizes the haplotype distribution among the NCL-affected and the clinical unsuspicious dogs. The chi-square statistic for testing these haplotypes for association with disease status in the Tibetan Terrier dogs was calculated as 5.5786 with degree of freedom 1, which had a p-value of 0.80, indicating no significant association.

**Table 2:** *CLCN3 *genotypes in NCL-affected and control dogs

Genotype	Tibetan Terrier					PON
	1/1	1/2	3/4	1/5	1/6	1/2
NCL-affected (n)	4		1	1		1
NCL-non-affected (n)	5	1		1	1	

^1 ^Genotypes correspond to the deduced haplotypes shown in Table 1.

## Conclusion

The presented data indicate that the detected polymorphisms in the coding and adjacent intronic regions of canine *CLCN3 *can be excluded as disease harboring mutations in the examined dogs. Therefore it seems to be likely that the entire *CLCN3 *can be excluded as a candidate gene for the late-onset NCL phenotype in Tibetan Terrier and PON dogs. As the candidate gene approach did not reveal the causative gene in Tibetan Terrier and PON dogs it might be indicated to perform a genome wide linkage scan using NCL segregating families to map the canine chromosome region harboring the deleterious gene.

## Methods

### Sequence analysis

The human reference *CLCN3 *mRNA [GenBank:NM_001829] was used as query in cross-species BLAST searches against the dog genome assembly (Boxer genome assembly 1.1). A single canine genomic contig of 577,638 bp was isolated [GenBank:AAEX01020012]. The human mRNA sequence was used to identify putative exons in the canine genomic sequence used for dog specific RACE primer design. Total RNA from lung tissue of a normal female Beagle (Biocat, Heidelberg, Germany) was used for amplification of RACE PCR products. Isolation of full length cDNA for the canine *CLCN3 *gene was achieved by a modified rapid amplification of cDNA ends (RACE) protocol with the FirstChoiceTM RNA ligase-mediated (RLM)-RACE kit (Ambion Europe, Huntingdon, UK). Briefly, in RLM-RACE uncapped RNAs were dephosphorylated before the cap of full-length messenger RNAs (mRNAs) was removed enzymatically. After this step an RNA oligonucleotide adaptor was ligated to the 5'-end of the decapped mRNAs. As only full-length RNAs carried a 5'-phosphate group, the adaptor was expected to ligate exclusively to full-length mRNAs, while the dephosphorylated other RNAs were not able to undergo a ligation reaction. RT-PCR using two pairs of nested gene-specific (Table 3) and adaptor-specific primer pairs (Ambion) were then used to amplify the complete 5'-end of the *CLCN3 *cDNA according to the instructions of the manufacturer. Similarly, the 3'-end was amplified using two pairs of nested gene-specific and 3'-adaptor-specific primers. 5'- and 3'-RACE products and an additional 1885 bp RT-PCR product using sense and antisense primers from exon 1 and 10 (Table 3) were cloned into pDrive plasmid vectors using the Qiagen PCR cloning kit (Qiagen, Hilden, Germany) and several clones were sequenced. The obtained canine cDNA sequences were aligned with partially overlapping canine EST sequences corresponding to the human *CLCN3 *[GenBank:BM537636,CF411209,BI398115,BU749098,BQ839554], and *CLCN3 *isoform e [GenBank:BM536915], respectively. The exact canine genomic structure was determined using the mRNA-to-genomic alignment program Spidey [14].

**Table 3:** Primer sequences for the amplification of canine *CLCN3 *cDNA

Primer	Sequence (5' – 3')	Localization within canine *CLCN3*	T_M_(°C)
5' RACE outer primer	TGTACGAGCCAGGACCTTCT	exon 4/exon 5 junction	60
5' RACE inner primer	TTTGTCATTTCCCATGCTGA	exon 2	60
3' RACE outer primer	TGCTTTAGTGGCTGCATTTG	exon 8	60
3' RACE inner primer	TGACTGTCTCCCTGGTGGTT	exon 10	60
CLCN3_F1	ATGGATGCTGCTTCTGATCC	exon 1	60
CLCN3_R10	CAGCAGCCAGAGTGGTATGA	exon 10	60

### Mutation analysis

Genomic DNA was isolated from a single NCL-affected PON dog, six unrelated NCL-affected Tibetan Terrier dogs, and eight unrelated clinical unsuspicious Tibetan Terrier dogs (> 8 years old). Clinical neurologic, behavioral, and ophthalmologic evaluations were performed on each dog by a single external consultant veterinarian [4,15]. The phenotypes of the affected animals have been confirmed by detection of autofluorescent cytoplasmic inclusions within neurons throughout the retina and brain after necropsy. The 13 *CLCN3 *exons with flanking sequences were PCR amplified and directly sequenced with the DYEnamic ET Terminator kit (Amersham Biosciences, Freiburg, Germany) and a MegaBACE 1000 capillary sequencer (Amersham Biosciences), using PCR primers listed in table 4 as sequencing primers. The association analysis for this paper was generated using SAS/HAPLOTYPE software, Version 2.1.39 of the SAS System for Windows (2003 SAS Institute Inc., Cary, NC, USA).

**Table 4:** Primer sequences for the amplification of canine *CLCN3 *exons

Forward primer	Sequence (5' – 3')	Reverse primer	Sequence (5' – 3')	T_M_(°C)	Product size (bp)
CLCN3_Ex1_F	AGCAGGGGTGGAAGAAATG	CLCN3_Ex1_R	AACTACAGAACCGCCCAGC	60	233
CLCN3_Ex2_F	ACCTAGTTCACCATTGTCTCTCA	CLCN3_Ex2_R	TATTTTGGCTGCCAGAGGTC	60	312
CLCN3_Ex3_F	ACCCCTTGCTCTCAAATCCT	CLCN3_Ex3_R	TTGTAGGGTGAAGGAGAGAACT	60	418
CLCN3_Ex4_F	GTCTCAACACTCCAAAAGTGGAC	CLCN3_Ex4_R	CTGTAATTAAACGGAGACTCATCTCA	60	321
CLCN3_Ex5_F	TGTGGAAGTAAGCCAAGAAACTC	CLCN3_Ex5_R	CTCCCCCTAAAGGCAAAAAG	60	318
CLCN3_Ex6_F	AAGTGTTCCTGTTTCCTGAATGA	CLCN3_Ex6_R	GACTGAGCAGTACTGGGGATG	60	459
CLCN3_Ex7_F	TTGGAAAGAGGTAGCCATCG	CLCN3_Ex7_R	GGCTTTTCTCAAGGTAAAGAACAT	60	936
CLCN3_Ex8_F	GCTGCAGCAAAAATTAGACCA	CLCN3_Ex8_R	AAATGGAACCCAAAAGATAAGAA	60	781
CLCN3_Ex9_F	AGTTTTATTTGTACTAGGATTTTGCTC	CLCN3_Ex9_R	CAATAGCAGTACTGTTTCATTTCTGTG	60	474
CLCN3_Ex10_F	TCCTGTCCTCCTTGACCAAT	CLCN3_Ex10_R	CCCCCAGAAACCCAACTAAT	60	579
CLCN3_Ex11_F	GGGACCAAATTCATGGGATA	CLCN3_Ex11_R	TGTTTTGGCAAAGATGTGGT	60	511
CLCN3_Ex12_F	GGACCTGGGATTTCGAACC	CLCN3_Ex12_R	TTATTCAGCAGGCATCTGGG	60	343
CLCN3_Ex13_F	ATCAAAGGATGGTTGCTGGA	CLCN3_Ex13_R	TTGCGATGTCGGAGTAACAG	60	647

## Authors' contributions

AW did the mutation screen and drafted parts of the manuscript. OD proposed the idea and was responsible for funding. CD performed the RACE experiments, analyzed the sequence data, and performed manuscript editing.
